# How Successful Are the Teams of the European Football Elite off the Field?—CSR Activities of the Premier League and the Primera División

**DOI:** 10.3390/ijerph17207534

**Published:** 2020-10-16

**Authors:** Kinga Ráthonyi-Ódor, Éva Bácsné Bába, Anetta Müller, Zoltán Bács, Gergely Ráthonyi

**Affiliations:** 1 Institute of Sport Management, University of Debrecen, H-4032 Debrecen, Hungary; bacsne.baba.eva@econ.unideb.hu (É.B.B.); muller.anetta@econ.unideb.hu (A.M.); 2 Department of Accounting, Institute of Accounting and Finance, University of Debrecen, H-4032 Debrecen, Hungary; bacs.zoltan@fin.unideb.hu; 3 Institute of Applied Informatics and Logistics, University of Debrecen, H-4032 Debrecen, Hungary; rathonyi.gergely@econ.unideb.hu

**Keywords:** sustainability, CSR activity, environmental protection, Premier League, Primera División

## Abstract

In the past two decades the sports sector has turned its attention to understanding the idea of sustainability, particularly to the practical steps related to this. The purpose of this study is to investigate the nature of CSR (corporate social responsibility) activities carried out by teams playing in the Premier League and the Primera División in the 2018/2019 season, and how these CSR actions serve environmental protection and society, manifesting the concept of sustainable development. We applied comparative analysis based on secondary databases. We examined the available reports regarding all the 40 teams, focusing on information about their CSR aspirations and related academic research results, and we worked out specific criteria to evaluate environmentally and socially related CSR activities. Arsenal and Real Madrid were chosen to show good practices that can serve as examples for the other members of the sports sector. At Premier League clubs, the practical application of the CSR activities has been intensively developed. Clubs share detailed statistical information about their actions, while some of the clubs even publish their future plans. The quantity and detail of the information found with Primera División clubs is rather varied. Some clubs introduce their CSR activities in full detail; however, in the case of most clubs, the accessible information is rather superficial and lacks any exact descriptions. The findings clearly show that the sports world is consciously shifting towards the realization of sustainable development, which requires a comprehensive reconnection of sporting society and an increase in awareness in order to achieve the efficient and successful integration of CSR activities into sport.

## 1. Introduction

The appearance of a responsible approach in the various sectors of the economic world may manifest itself through a number of activities. One of these—today an increasingly popular research field—is the study of the responsible approach of the sports sector [1]. The increased interest in this topic is manifested through research that looks at a number of issues. Even more academic research analyzes the motives of CSR involvement [2,3], communication [4,5], the decision-making process in connection with CSR [6,7], financial outcomes [8], or CSR-linked sports sponsorship [9,10].

Investigating the CSR activities of sports clubs and leagues, especially related to football, is a popular research area within professional sports [11,12,13,14,15,16]. These research studies examine the environmentally and/or socially related CSR activities of the clubs in a league, or of a specific club. However, very few studies compare the measures of all clubs in several leagues, covering both areas of CSR.

The purpose of this paper, therefore, is to fill this gap by analyzing the CSR activities carried out by clubs playing in the Premier League and the Primera División in the 2018/2019 season, and how these CSR actions serve environmental protection and society.

Football is one of the most popular sports in the world; thus, it provides a forum for connecting the passion of the game to the endeavors of individuals by encouraging them to take part in various CSR movements, transforming all the factors of CSR into a service for society [17] and the environment [18]. As attention to social responsibility is increasing, sport can be seen as an opportunity to make the quality of life better and function as a link between social, environmental and economic needs [19]. On that basis, the football world, both nationally and internationally, has begun to take responsibility for their societies by way of emphatic CSR measures, programs and initiatives to support their communities and protect the environment.

In accordance with the above, this study aims to find answers for the following research questions:

1. What kind of CSR activities are football clubs involved in?
2. Which environmental fields are targeted by football clubs?
3. What activities supporting the development and well-being of society occur at the clubs?

We also find it important to present a few good practices for each league, without aiming for an exhaustive coverage.

This paper is organized into six sections. The introduction is followed by a section interpreting CSR in sport, clarifying the meaning of socially and environmentally related CSR activities and the opportunities for CSR in football, before providing the contextual background for the study. The third section is devoted to a more detailed account of the method used. The paper then goes on to present the CSR activities of the Premier League and Primera Division clubs. The fifth section evaluates the results, before drawing conclusions.

## 2. Literature Review

### 2.1. Corporate Social Responsibility in Sport

The origins of social responsibility have been linked to various historical events by each field of the technical literature. To define what a “responsible” company means is a complex thing to do as the technical literature has diverse opinions. The concept has been developing and forming since the 1930s. However, all these definitions are unanimous on the major points: the aspiration to enhance a healthy and sustainable balance of economic interests, social requirements, and environmental limits throughout all business activities. We can read in a number of studies [20,21,22,23] that the essence of the concept is that companies integrate social and environmental concerns in their business operations and in their interaction with their stakeholders on a voluntary basis. That is to say, complementary goals based on moral values should be included in the set aims; this tendency could lead to the concept of a “value-based” or “responsible” corporation. This means that a company has environmental and social responsibilities, and accordingly, throughout its actions—as if using a filter—“makes rational choices from morally acceptable alternatives” [24] (p. 48). In other words, when forming objectives, the interests of all other parties need to be taken into consideration.

However, it should be mentioned that the 1970s already saw a serious debate about the business importance of CSR. Milton Friedman stated that companies are only liable to their direct investors [25]. In his view, CSR and business are two completely independent areas that should not be confused, as it is not the business’s job to deal with social problems; this is rather a duty of government.

Questioning the exclusivity of “implementing CSR as an ethical behavior”, several studies have addressed additional motivations for the introduction of CSR in recent years:

- CSR as a response to social criticism: Many companies have only begun to address CSR in response to attacks and scandals. True CSR can be an integral part of corporate culture; it is not advisable to take a defensive stance against the social environment (“stakeholders”) “in a hurry”, but rather to be partners with them [26].
- CSR as productive factor (the business case for CSR): This consideration suggests that a morally “good” business can become an economically “good” business in the long run. In this sense, CSR is nothing more than a kind of corporate self-interest, a long-term profit factor [27]. Consistent with this, after a while a firm with a well-established reputation can expect a strategically competitive advantage as it increases its CSR engagement and activities. CSR represents a major criterion used by institutions in making their investment decisions. According to Campbell [28], firms engage in CSR activities because they are in contact with several actors in the environment. Institutional investors prefer firms with a better CSR ranking over those with low rankings. Consistent with this argument, better CSR performance indicators are much more attractive to the institutions holding shares in a company. Institutional investors can then use these connections to turn corporate social engagement to their advantage and make it profitable at the same time. In connection with the sports industry, we can say that the interest in CSR and stakeholder management has become the focus of attention these days, as well [29,30].
- CSR as a business constraint: Often, partner companies have CSR expectations of companies.
- CSR as a marketing tool: CSR can often be an effective marketing tool, as a socially responsible corporate image can help a firm to enter the market, strengthen its market position [31], and generate demand.
- CSR as a source of innovation: Despite the growing popularity of CSR, only a small proportion of companies have embarked on this path, so a CSR strategy can still be a very good tool for gaining a competitive advantage [32], to create a self-image so a company can differentiate itself in the market.
- CSR as a fashion: Nowadays, a socially responsible corporate image can be fashionable; the media also favors this approach. CSR as a fashion can manifest itself in consumers being willing to buy from responsible companies or in investors being willing to invest in proven responsible companies.

In addition to these aspects, we can also mention advertising, public relations, publicity, sponsorship, and word-of-mouth as the main methods of promoting CSR activities [33].

We can see that the motivation behind the introduction of CSR can be diverse and mixed in practice, and that actors from the sports sector cannot be an exception to this.

In the last decade, the number of international articles and work on sports science which examine the spread of CSR activities carried out by sport organizations (companies and clubs) and non-profit corporations, as well as large scale sport events, has grown dramatically [34,35,36,37,38].

Sport has an extraordinary unifying power in terms of eliminating cultural, social, ethical, and religious boundaries, and contributing to the formation of a modern international market stretching over borders [39,40].

The unique role of sport in society and the recognition of the opportunities in sport have made it clear that integrating sport and CSR has immense possibilities.

We can summarize in seven points how sport can serve CSR [19,38,39,41]:

- As sports are popular and reach people around the globe, sports CSR activities receive mass media attention and provide access to viewers with their power to communicate.
- Sport CSR appeals to young people. If a CSR activity is connected to a sports organization, team, or personality, young people are more likely to become involved in it.
- Physical exercise provides a forum for organizing excellent programs and initiatives that can result in positive health impacts. Sport CSR is a brilliant means for this purpose, too.
- Sport CSR requires constant group participation and therefore makes a strong contribution to social interaction.
- Cultural understanding, tolerance, and integration is another powerful element that characterizes sport CSR.
- There are certain sports which may lead their participants to environmental and sustainability awareness.
- Those who take part in sport CSR activities immediately experience their beneficial emotional effects, as well.

We can see that the abovementioned features are related to the social and environmental aspects of CSR, so sport is capable of supporting both aspects.

According to Yoon and Chung [42], (1) external and (2) internal activities need to be differentiated from the social aspect. External social responsibility accelerates the positive effects of organizational activities and operations on society, the economy, and the natural environment [43]. These activities target constituencies and communities supporting teams and leagues or other communities in need. As opposed to this, internal social responsibility focuses on the organization itself: to improve the well-being of the workforce, their lives, and productivity and profitability. External and internal initiatives reflect upon and accelerate each other. This social sustainability aspect brings about and leads to the realization of equal opportunities, diversity in the workforce, the creation of connecting points to the surrounding community, a path to better life quality, and a democratic approach.

Together with environmental guidelines, environmental sustainability is increasingly dominant in CSR, not only for recreational sports companies but for all sport organizations too [14]. Academic work is currently focusing on sport and the environment, and this interest has grown steadily over the past 15 years [44].

As a result of environmental awareness, environmental issues have become valued and mainstream. Prominent sports organizations and clubs have green management as a priority and apply a green marketing strategy, too [45,46]. These tools are expected to lead their companies to sustainability and allow them to pursue environmental values. With a CSR based way of thinking, companies will introduce environmentally responsible business practices, such as energy, waste and pollution reduction, and a decreased use of toxic materials.

It is unquestionable that all sports need natural resources to some extent, and have an impact on the natural environment, requiring land and affecting the natural landscape. Several animal and plant species are becoming extinct, and huge quantities of water are contaminated. Moreover, using non-renewable materials, the waste emissions in the building and operation of sports facilities, travelling to and from these places, catering, health care provision, commercialism, and the production and disposal of sports equipment cause the kind of problems that CSR needs to face [37,47,48].

In modern society sport is increasingly popular [49]. More and more bodies connected to sport have been pioneering new strategies to integrate the environment into their business strategy [50], so sport is a substantial enabler of sustainable development [51].

According to Wilson and Millington [52], all people, activities, businesses, or organizations involved in producing, facilitating, promoting, or organizing any sport-related business or product follow, intentionally or unintentionally, environmental principles:

- Acknowledging that sports-related activities might have a damaging impact on the natural environment, actors in the sports sector are positioned to contribute to, or even lead, a response to sports-related environmental problems [53].
- To adopt environmental principles involves committing to work with other stakeholders who are also concerned with environment-related issues.
- Innovation and technology-oriented solutions are crucial when dealing with environmental problems in order to maintain the most sustainable facility possible.

However, Miller [54] sharply criticizes the green efforts of the sports sector. In his view, one way of achieving economic gain behind moral good deeds is the so-called “greenwashing”. According to this view, “greenwashing” is the process of conveying a false impression or providing misleading information about how a company’s products or activities are more environmentally sound.

It is clear that even today the motivations behind the use of environmentally related CSR activities are often unclear. However, it is certain that changes are needed in several areas in order to make the operation of the sports sector greener.

According to Shiply [48], to make sport greener, the following points are necessary:

- To have representatives of sport and those promoting nature conservation and environmental protection working together;
- To work out functional guidelines for sustainable development in sport;
- To support and further develop types of sport that are compatible with nature and the environment;
- To make sports-related infrastructure more environmentally friendly;
- To ensure strict compliance with environmental standards by government and agencies financing sport facilities;
- To integrate environmental management into the operation of sports administration, clubs, associations, and commercial sports operators;
- To grant priority to voluntary commitments for achieving conservation aims.

It can be clearly seen that it is necessary for sports society to unite on a wide scale, so that CSR activities serving our society and environmental protection can be effectively and successfully integrated into sport.

### 2.2. Corporate Social Responsibility in Football

It has been stated that football is one of the most popular sports entertainment activities in the world, with a great potential for future development [11]. Professional sports clubs in Europe and major league sports in North America [16] are amongst the pioneering organizations increasingly integrating CSR [40] into their operational and strategic tools to achieve their goals in the changeable atmosphere of competition, while constantly keeping sustainability in mind, as well.

Since the late 19th century, people have understood the importance of sports communities and appreciated the success of professional football clubs. It has been discussed by [55] that sports play a unique role in society and are able to address problematic social issues in various situations. A good example of this is Celtic FC, when, around 1888, the club recognized the potential of the fans’ pride in their team to remedy socio-economic inequalities in society. As a result of this, Celtic FC has developed a unique leadership system allowing their fans to have a say in the organization of the club [56], which we see now as an early form of CSR activity [57].

Today, more than a 100 years later, football clubs have changed profile, growing from local organizations providing a meaningful way of spending time for communities to large-scale, commercialized enterprises. A great deal of CSR activities occur at present in the sports industry, especially in football [16], and according to another study [58], these programs are organized at all levels (international, national, regional, city), mainly in the city or the town in which the football club resides and largely operates.

Improvement in CSR has been particularly flourishing in the case of the best clubs from the so-called Big Five—England, Spain, Germany, Italy, and France—which have already become global brands and have fans all over the world. Therefore, the clubs are expected not only to achieve their ambitions in sporting terms but also to reach certain economic goals, depending both on their ranking and their budget. In addition, football clubs have turned into organizations that not only serve as a source of entertainment but are also able to promote specific values (e.g., an environmentally conscious attitude) and pursue several social goals to improve local communities’ quality of life [59].

In the previous chapter, we saw that there may be a number of motivations behind the introduction of CSR. However, it is an indisputable fact that world-leading football clubs which reach out to the general public, must always take responsibility for their actions. By their operations, clubs implicitly (and even explicitly by their communications) indicate which stakeholders they consider important, whose interests are taken into account, what social role they intend for themselves and how they become involved in social life and solve environmental and social problems. All of this significantly determines the perception of clubs. In our view, these factors are also crucial when a club is considering implementing CSR measures, and it is very difficult to state clearly whether all this is done exclusively by the club at a compliance level or pro-actively.

## 3. Materials and Methods

### 3.1. Study Design

The aim of this present study is partly to examine whether in the 2018/2019 season the teams participating in the two selected leagues made their CSR activities public and partly to analyze what sort of measures were taken in their responsible approach. During our research, we were looking at the following questions:

1. What kind of CSR activities are football clubs involved in?
2. Which environmental fields are targeted by football clubs?
3. What activities supporting the development and well-being of society do the teams take part in?

We also find it important to present an outstanding good practice for each league in detail, which will give us a full picture of how the sports sector, more precisely a football club, can contribute to manifesting sustainable development.

### 3.2. Sample

The focus of this study is on the clubs that took part in the Premier League (England) and Primera Division (Spain) in the 2018/2019 season. The reason for choosing the two leagues was based on the selection of the two most watched football leagues in the world and on the monitoring of the operation and measures taken by the clubs across a wide range of society. These factors can help clubs get their message and their positive example across to millions of people. With this synergy effect, the commitment and sensitivity of sports society to responsibility can be increased, and their conscious behavior can be developed.

The Premier League is the highest level of the football league system in England. Maintained by 20 clubs, it is based on a system of promotion and relegation with the English Football League (EFL). The competition was established as the FA Premier League in 1992 after the decision of clubs in the Football League First Division to continue working independently of the Football League founded in 1888, and to take advantage of a profitable television rights deal [60]. The Premier League is the most viewed sports league in the world: It is available in 212 territories to 643 million homes and reaches a calculated TV audience of 4.7 billion people [61]. Today, England’s top domestic league is one of the most popular and richest sports leagues worldwide, with six of the ten richest football clubs in the world as of 2019 [62].

In Spain, football is the sport with the highest number of registered players and clubs among all Spanish sport federations, according to data provided by [63]. The First Division (Primera División), well-known around the world of football, is La Liga. The Primera División was founded by 10 clubs in 1928. The First Division includes 20 professional teams. Like the Premier League, it is extremely popular, with an average attendance of 26,933 for league matches in the 2018–19 season [64]. As UEFA’s league coefficient rankings show, La Liga has been the top league in Europe in each of the seven years from 2013 to 2019 (this has been calculated using accumulated figures from the five previous seasons) and has topped Europe for 22 out of the 60 ranked years up to 2019, being the absolute winner [65]. Table 1 presents the two leagues with the teams studied in this research.

When introducing good practices, we chose a team from each league which pursues a wide range of CSR activities and can serve as an example to follow for other football clubs. We selected Arsenal FC from the Premier League and FC Real Madrid from the Primera División.

Real Madrid has always been a member of the Primera División and has never been eliminated from the championship. With their 33 champion’s titles and 19 cup victories, they are the most successful Spanish team. In addition to this, they have won the Champions League 13 times and the UEFA Cup 3 times. According to the listing of Forbes magazine, Real Madrid is the largest and most highly valued football club, managing the second highest revenue: Their budget is around 840 million euros, while the full value of the club is more than 4 billion euros [68]. Real Madrid is a founding member of FIFA and the European Club Association.

Arsenal FC is a football team from the Holloway district in North London. It is one of the most successful teams in English football: It can boast 13 First Division and Premier League Champions titles and 13 FA Cup titles. The club was established in 1886. Over the years, the club has changed its headquarters several times, arriving at their final home, the Emirates Stadium, in 2006.

### 3.3. Content Analysis

During our study, we applied comparative analysis based on secondary databases.

In order to find answers to our research questions, we examined freely accessible data, and all the documents at hand about all teams with special regard to their CSR activity. We overviewed annual reports, sustainability/CSR reports, information presented on the websites of the teams, academic research, data, and articles concerning the teams. We contrasted our findings with those of international organizations (e.g., the European Football for Development Network, the European Club Association) which also deal with this topic.

In addition, CSR actions are often implemented via foundations, which have become the standard channel for football clubs which deploy CSR. The appearance of foundations can be related to the strengthening of clubs’ CSR activities, as they operate either as a separate organization or as an independent organizational unit within the club. In the past, responsibility appeared in clubs mainly as part of an organizational unit. We can conclude that the foundation model has enjoyed great popularity in the football sector, as well [16,69,70]. If the clubs had such foundations, we checked the homepages of the foundations, as well.

#### 3.3.1. Environmentally Related CSR Content

When analyzing CSR initiatives, the study aspects need to be divided into two parts, because—as we have seen in the definitions of the technical literature—the concept of CSR focuses on two areas: environmentally related and socially related CSR activities.

As we were developing the evaluating criteria for examining the environmental aspects, we relied upon work by researchers who are specialized in the field of environmental effects and the challenges faced by sport [14,37,52,71,72,73,74,75].

According to these researchers, the impact of the sports sector on the environment can be interpreted on the basis of the following seven aspects:

1. Environmental conditions;
2. Land use and landscape;
3. Resource management and use of energy;
4. Waste management;
5. Environmental pollution;
6. Protection of cultural heritage;
7. Environmental impact of activities deriving from sport (operation of facilities, transport, accommodation, sports tourism, food, office work).

The literature on the relationship between sport and the environment is consistent in that the 7 factors mentioned above are mentioned as key environmental elements and will be examined in detail. There may be a difference in the depth of the studies concerning each factor or in the inclusion of a new aspect within each factor.

We surveyed—according to the findings of academic research—in which sectors of sport intensive environmental awareness raising and the development of a responsible approach are necessary. Subsequently, the 7-factor criteria named by the literature on the environmental problems of sport were modified/concretized to the criteria considered most appropriate for our own research (i.e., a review of environmental efforts related to the activities of football clubs).

Eventually, we named the following six areas, on the basis of which we can classify the green activities of a sports organization (here understood as sports clubs) and the environmental protection efforts made during the building and operating of the sport facilities and the organization of sports events:

1. The use of land and landscape (e.g., conserving biodiversity, protecting the ecosystem, largely modifying an entire region);
2. Clean energy (e.g., applying renewable energy, avoiding climate change, reducing carbon footprint);
3. Energy efficiency (e.g., using clean technology, LED lighting, energy saving, movement sensors, voltage optimization equipment),
4. Water management (e.g., rainwater-collection system);
5. Waste management (e.g., prioritizing the 4R principles: reduce, reuse, recycle, repair);
6. Environmental impact of activities deriving from sport (e.g., transport, food, office work).

In this study, we have been searching for answers to questions related to these 6 areas from the information released by the clubs.

#### 3.3.2. Socially Related CSR Content

To study and value socially related CSR activities is not easy because these are very diversified. [76] mention the following activities: charitable donations (philanthropy); fundraisers (“half of tonight’s concession proceeds go toward…”); athlete and employee participation in community programs; social media promotion; program development for causes (e.g., improving literacy, improving youth education, improving health); internal league training programs (diversity, violence prevention, life skills, etc.); internal safety programs.

In other studies [43,77,78,79,80], socially related activities are grouped into external and internal operations. External activities are included but not limited to the following: efforts involving physical activity; building sports infrastructure in underserved areas; fighting racism and other forms of social injustice, ways to fight poverty and extend working opportunities, cooperating with nonprofit organizations supporting health issues or causes that enhance education, such as attending university or trade school.

As far as external activities are concerned, it has been shown by a global poll that the external efforts that seem to be most popular among fans are those that support children, health, and the communities in which the teams and leagues operate, and connect to the sport sponsoring the effort.

Internal activities include the following: making sure athletes and other employees are as safe as possible; are trained in life skills and issues of social awareness like domestic violence, financial planning, racial and religious sensitivity, education, family responsibility, and other issues; are deeply against racism and other forms of prejudice within sport; and agree with diversity.

For the sake of making the activities of the examined 40 teams as systematizable, transparent, and comparable as possible, we followed the criteria of research where socially related activities are organized into four major aspects [5,16,81]: (1) educational programs, (2) sport/health programs, (3) social/cultural programs, and (4) charity programs.

#### 3.3.3. Case Study

Case study research has long been applied to contribute to research in sociology, business, and anthropology. A qualitative descriptive case study methodology was chosen because it is a complex tool for researchers to examine complex phenomena within their contexts [82]. When the approach is applied appropriately, it becomes a valuable method [83]. It is the opposite of quantitative methods, as they mostly examine a few previously selected qualities [82]. We almost completely sacrifice representatively for complexity, since we only examine one single case with all its complexity and uniqueness. Two substantial peculiarities of a case study are complexity and conceptuality [84]. Complexity means that we present the case studied in its complexity. However, in conceptuality, we examine and show cases not only in themselves, focusing on their specific qualities, but in cohesion with the environment they operate and act in.

The case study method concentrates on the significant and complete data necessary to characterize real-world events [85]. This method and design are appropriate for studying the use of CSR within a sports club. Selecting the two clubs, we used a purposeful sampling method.

The first step in selecting the clubs was to compare socially related activities. We wanted to choose clubs that present their programs to a wide range of society in as much detail as possible, based on as much recent information as possible. We then compared the environmentally related activities of the clubs we had thus narrowed down (Premier League: Arsenal FC, Chelsea, Manchester United, Manchester City; Primera Division: Atletico de Madrid, Barcelona, Celta Vigo, Real Betis, Real Madrid). Finally, we selected one club per league, in which, based on our evaluation criteria, environmental measures are applied in as many areas as possible and this is made public in sufficient detail (plan-fact data, financial data, future plans, development opportunities). Taking these into account, Arsenal FC and FC Real Madrid were selected because these two clubs present their CSR activities in both areas in the most detail.

## 4. Results

### 4.1. CSR Activities of the Premier League’s Clubs

The environmental related CSR activities of the clubs in the Premier League can be seen in Table 2. (x: club does activity in this area; -: club does not deal with this area; neither X nor -: we did not find any information).

Based on the results obtained, it is clear that energy efficiency, water efficiency, waste management, and transport are the areas that all teams are working on to make greener. This is understandable as, for example, the operation of stadiums, the care of an appropriate pitch, the uninterrupted provision of broadcasts, and the care of spectators on match days all come at a huge cost. Making these areas more environmentally friendly can also bring an economic benefit. There are areas where we have found information about greening for a few clubs; however, we believe they are important areas in greening a club; they cannot be ignored and may receive more emphasis in the future, providing a way forward for even more environmentally conscious operations. In the following, we present our results by area.

It is clear that land use and landscape were only relevant for two clubs, according to the documents analyzed. The clubs report on regeneration and land alteration projects in order to create their training centers based on environmental protection values.

As for clean energy, we found that one club does not take part in any activities. For 6 clubs we did not find any information on this. In the case of all the other clubs, establishing solar panel systems, or installing air source heat pumps are common ways of reducing their carbon footprint. Energy efficiency appears to be emphasized at all clubs, in most cases through LED lighting (inc. floodlights) and high-efficiency building services systems and intelligent controls. Water efficiency is present in all but one club, and there was another club without any available data on the issue. In the other clubs, the most often mentioned operations are rainwater collection used for irrigation, dirty water recycled and used for irrigation, waterless urinals, and low-flow fittings and fixtures. All clubs took steps in waste management to contribute to environmental sustainability (including food waste used for anaerobic digestion; composted organic waste; creating battery recycling points; and sending plastic, cardboard, wood, paper, and aluminum for recycling). Five teams have already received a kind of environmental certification, which proves that they genuinely make an effort to sustain environmental protection.

In connection with the environmental effects of activities related to sport, we highlighted 3 areas: transport, food, and office work. We only found three clubs where going green in office work has appeared (e.g., using recycled paper, selective waste collection, energy saving lighting, using personal cups instead of plastic ones). Two clubs are not concerned with plant based and low-carbon food activities, and there was one club without any relevant information on the topic. All clubs make efforts to reduce the strain supporters put on the environment by travelling around: the clubs promoted and sponsored public transport and established bicycle lanes and footpaths. The socially related CSR activities of the English clubs were put in four main categories, as shown in Table 3.

In the case of socially related CSR activities we found almost the same results, although a difference can be observed in the number and diversity of activities related to each area. According to the documents analyzed, it was seen that the clubs provide programs from childhood to adulthood through their diverse projects, with a difference in the emphasis on different areas.

Educational programs are very popular at all clubs. An important element of these programs is to raise children’s awareness to see sport and school life both as a place of success, to view them with equal importance as things which help their learning processes in various subjects and develop their skills. In several clubs, high school age groups (16–19) are offered technical programs.

A vital area of sport/health programs is the increase in physical activity for all age groups (e.g., 50+ walking football). Clubs are trying to reach children: They hold sports days, they organize training sessions and cups in sports other than football for both men and women, and they provide health preserving programs in workplaces with trained coaches. Mental health preserving activities, health checkups, and the recommendations of dieticians are available in more and more clubs. One of the most influential actions of the social/cultural program is that clubs establish “schools” worldwide (e.g., Africa, Indonesia) the aim of which is to take up underprivileged young people and help them to integrate into society through sport. Disability sport is also present in all clubs, which is part of the social/cultural program. In most clubs, sports training sessions are held for them weekly, they visit development facilities to see these young people and hold tournaments for them, too.

Charity programs are also present in almost every club, although for two clubs there is very little information on this issue. No exact description of their activities is available. The other clubs report on their involvement in subsidizing organizations and programs. These can be money/equipment/food donations.

### 4.2. CSR Activities of the Primera Divisón’s Clubs

Table 4 reviews the environmental activities carried out by the clubs of the La Liga.

In the case of the Spanish clubs, it can be said that less activity is observed compared to the English clubs in each area, based on the documents analyzed. Most of the clubs make environmental efforts in the area of waste management, but measures can also be observed in the areas of energy efficiency and water management. A quarter of the clubs also implement land regeneration protection programs, but for example, there are hardly any measures in the area of the environmental impact of activities deriving from sport.

We can see that land use is significant among the environmental protection activities with five clubs. In this area, we can read about the creation of training centers and their surrounding areas, tree planting with the purpose of providing better air quality, the reduction of pesticides and other chemicals and how to handle protected species in the area. With six clubs, we can read about efforts in connection with clean energy and another three clubs are concerned with increasing energy efficiency. In these two fields, the following steps are the most common: CO_2_ emissions reduction with the help of efficient technology, LED lighting, incorporating photovoltaic panels for the generation of renewable energy, optimization of the air conditioning system, and the use of solar energy to heat water.

Seven clubs are involved in improving the efficiency of water management. Most of them use both rainwater and greywater in order to reduce their demand for municipal drinking water. Recycled rainwater is later used to irrigate the pitch and clean the stadium. They try to minimize the risk of flooding during extreme weather conditions. Eleven clubs find it important to deal with waste management: They have signed several-year-contracts to carry out waste collection and paper and glass recycling. More than one club point out that they are reducing single use plastic.

Real Madrid, Celta Vigo, and Athletic Bilbao have a well-known certification (LEED, ISO). FC Barcelona is the first sports complex in the center of a major city that has obtained the German DGNB pre-certification, a system for evaluating and certifying sustainability, which considers the environmental, economic, and cultural aspects of the buildings. Real Betis has officially joined the Climate Neutral Now initiative of the UN Climate Change, committing to take action to reduce its greenhouse gas emissions. Two clubs mention among their actions that in their operations creating sustainable transportation is important both for the clubs and their staff and for the supporters. They try to raise supporters’ awareness about using public transport, riding bikes, and walking. None of the clubs mention environmentally friendly action in connection with eating.

Real Madrid is the only team involved in ecological action related to their office work. They are doing their best to inform their workers on this subject; workers can drink filtered water instead of tap water, and employees have been asked to reduce their plastic bottle use.

Table 5 shows the socially related CSR activities of Spanish clubs.

In the field of socially related CSR activities, similar activities can be observed as in the case of English clubs.

In connection with educational programs we can say that for only one club were we unable to find any information about it. Sport/health programs are significant with all clubs. These two areas are closely related in their operation. Clubs have a strong interest in promoting sport in general and football in particular as a means of encouraging education in values and encouraging healthy habits in children and youngsters. In order to promote sport, healthy lifestyles, and learning, players visit schools at several clubs. They also organize seminars, conferences, and thematic lectures for the employees in the abovementioned subjects. There are clubs which promote innovation in sport in various fields such as medicine, physical education, sports law, and technology.

It was more challenging to find information about social/ cultural programs. Clubs list among their goals the fight against social exclusion and take on underprivileged young people through sport.

All clubs mention charity work; they regularly donate tickets, food, and money; they support disability sport, and in relation to the present pandemic crisis, they have also donated protection garments to health facilities.

### 4.3. Case Studies

#### 4.3.1. Arsenal FC

Arsenal FC is one of the most dominant clubs in the English first division. In the past few years, the club has recognized that they can have a significant role in manifesting sustainable development by integrating activities serving society and the environment and pursuing responsible attitudes. Apart from environmentally related CSR activities, information about social activities has also been found in their reports concerning the subject, on the club’s website, and in other publications. We present the socially related CSR activities according to four aspects, and underline their major actions in order to give a full picture of the principles of each area:

1. Sport: The club has always incorporated sport in their community work, and by expanding it further, young people have become more and more involved. Arsenal began their activities at Highbury as well, providing more than 50 sport training sessions to over 30 schools [98]. Women’s football is also emphasized in the life of the club. It was Vic Akers who founded Arsenal Ladies out of the Arsenal in the Community team in 1987. Ever since, they have been known as the most successful women’s team in England [121]. Arsenal Ladies take on a huge share of the outreach work: They visit schools, do community projects, raise awareness, and encourage participation in the women’s game. The club promotes other sports than football as well. A good example of this is the hockey program established in the 1980s and the bowling session that still operates today. In addition, Arsenal in the Community (the overall name of their social program) is also involved in the multi-sport delivery program, the Premier League 4 Sport. It aims to encourage hundreds of young people to take an active part in doing Olympic sports. With significant results in badminton, basketball, handball, hockey, netball, judo, table tennis, and volleyball, Arsenal in the Community—supported by excellent local coaches, schools, and clubs—works on creating a network as the place for talent and skills to evolve and friendships to be made [120]. During a review of sports programs, it was seen that by promoting other sports besides football, they also try to make sports attractive to both boys and girls, which may be important in shaping the health behavior of young people.
2. Education, employment, training: Within this area, we will highlight a few educational programs. Arsenal has a special, novel education program designed to fuse football and learning: The Arsenal Double Club. Just like the two halves of a game of football, the idea starts with a 45-min classroom session, where participants are presented with Arsenal-related educational resources. This is followed by a 45-min football coaching session. The Double Club scheme was originally planned as an intervention program, and over the years, it has grown to include a number of academic subjects, such as science, healthy living, literacy, modern languages, history, business studies, and geography. The Enterprise Program was started in 2011. It is a branch of Arsenal in the Community, which make careers advice available to students in north London secondary schools about the various jobs available at the football club. The training is followed by practice and firsthand experience [122]. With the help of the trainee programs, young people have been given a chance to get coaching qualifications and job opportunities since 1985. They have a number of these trainees working full time for the club and a wide range of jobs locally. Their two-year course allows students to work as football coaches or PE teachers: it is called the Arsenal Football and Community Sports Coaching Foundation Degree [116]. The Arsenal Gap Program is designed to coach young people “the Arsenal way” locally and overseas. Once Gappers obtain their certificate, they are sent to local schools to experience coaching practice, and then, they can travel abroad to work for one of the international Soccer Schools or community projects of the club. The scheme can boast 157 people as participants working in more than 120 schools in 16 countries around the world. Beside operating international programs, they also pay attention to local communities giving employment opportunities to people in Islington, Camden, and Hackney. Through the Arsenal in the Community courses, people can get qualifications in 12 weeks that can contribute to getting a job. When compiling educational programs, special attention is paid to maintaining the balance between sports and school, and after basic education, providing young people with further training and practical opportunities so that they can appear on the market as members of a well-trained workforce, thus providing them with a livelihood.
3. Social inclusion: The Arsenal in the Community program has provided social involvement for many since 1985. The program that focuses on this cause is called Arsenal’s Positive Futures and Kicks [116]. A joint venture has been funded by the Arsenal Foundation with the purpose of rehabilitating and medically helping survivors of torture. It was jointly founded by between Arsenal in the Community and Freedom from Torture.
4. Giving: One of the initiatives the Arsenal Foundation promoted was that they invited two programs to Islington for the first time: Save the Children’s “Eat, Sleep, Learn, Play!” (ESLP) and “Families and Schools Together” (FAST). It made a difference to the well-being of more than 100 families locally. They donated £70,000 to this cause [117]. To improve the position of disabled sport, Arsenal Foundation donated £5000 to the British Wheelchair Sports Foundation. Through the project 250 children and 150 adults will be introduced to a variety of different sports. All of the people involved have a physical, sensory, learning, medical, or mental disability [119]. Several fundraising events are held across the season by the Foundation which aims to encourage everyone connected to the club to be active in charity work. The highlight of the annual fundraising is the Arsenal Foundation Ball, which is held following every season at the Emirates Stadium. In 2014, more than £325,000 was raised at the Ball to subsidize several projects aimed at young people [116]. The club is present not only in the surrounding regions, but also in quite a few international projects in the past few years. Save the Children, which has been the global charity partner of the Arsenal Foundation since 2011, is a leading independent organization that fights for the rights of children all over the world [123]. Arsenal also partners with Save the Children’s charity work to support disaster-hit areas like Japan, with its earthquakes and tsunamis, or East Africa, Syria, the Philippines, or Nepal. Arsenal set aside another £300,000 and provided it for Save the Children in order to help out in Indonesia in 2013. In their charitable programs, they place great emphasis on the basic units of the local community, supporting families and children, but they have also been involved in international projects in recent years.

The club has recognized that its activities put an intense burden on the local, national, and global natural environment. As a result, they set themselves a target not only to keep the environmental guidelines and threshold limits but also try to anticipate the requirements, both in the case of the operation of the stadium and office work. Since the beginning of the 2000s, more and more activity has been observed in their green efforts. They are working on the implementation of an environmentally friendly project covering more and more areas, which has both environmental and social and economic benefits.

The activities they have carried out for environmental protection are presented below in seven points:

1. Selecting an area, the construction: The home of the club has been in London’s Islington district since 1913. A demand for a newly built stadium emerged in 1999 and with it a new initiative aimed at doing something about the local environment. With the building of the Emirates Stadium, the reconstruction of the whole surrounding area over 25 hectares began. The area reconstruction program, which was the major project of the development, cost 390 million pounds. The restoration project related to the stadium was the largest regeneration project in Europe. As the first step of the project, abandoned industrial and waste management buildings were pulled down, in the place of which a new waste recycling center was established (60 million pounds). Not only did it serve Islington but also the recycling aspirations of the surrounding districts. They were involved in building industrial parks, a road network, bicycle lanes, parks, and green areas and paid special attention to intensified public security. The stadium was handed over with 60,361 seats in July 2006. Through the reconstruction program, over 2600 new jobs were generated with 1800 of them long-term positions. For a nominal amount, 2500 new or restored buildings were sold [87]. It is clearly visible that the land regeneration program touched all the three pillars of sustainability. The projects realized in environmental and social areas brought about significant economic benefits within a few years, which resulted in the appearance of a number of partner companies in the district.
2. Waste management: The emergence of sustainability principles can be detected not only in the construction of the stadium but also in its maintenance. The club became aware of the fact that sustainable waste management must be a key factor in its environmental strategy. With this goal, the amount of used materials is reduced, and the club do their best to avoid using materials that are not recyclable and aim to use recycled resources in the first place. They also set out incentives for their suppliers to be conscious about and reduce their impact on the environment. During the matches, they make a special effort to collect plastic cups and bottles in separate containers. On match days, 80% of the waste was recycled and with the help of LED lights, energy consumption was reduced by 31%. Consequently, 7 million kg of carbon emissions were avoided [92]. In October 2007, a carboard box compressor—a baler—was bought, which helps them to better prepare the waste for transportation. The cardboard boxes and pallets are recycled by the transporting company. Afterwards a large-scale waste container was installed for storing large amounts of waste. An estimated 10 tons of cardboard and plastic bottles are recycled monthly [93,180]. This machine is used for compressing materials from all the catering units, shops, and broadcasting facilities [93]. During matches, an average of 32,220-litre rubbish bins are filled with glass, which is equivalent to 1.5 tons of glass being recycled [87]. The used oil produced by catering units (snack bars, restaurants) is transported by a partner for biodiesel production purposes. Food scraps are composted. Food and grass is composted for Islington residents to use. In community projects, food is donated to local food charities. Old furniture is given away to local sports and community clubs. The Emirates Stadium’s old floodlights were also released to sports clubs. Pallets are reused by the supplying company as a rule [86].
3. Renewable resources: In the Premier League the Emirates Stadium is the first to cover 100% of its energy demands for its operation from renewable resources [90]. In the United Kingdom the club was the first to install a storage cell system at the stadium. The system is capable of generating enough energy to supply the stadium for 90 min, thus the Emirates Stadium is able to run on this storage cell generator alone during matches. This energy equals the 2-h energy demand of 2700 homes [89,91,92]. These storage cell accumulators play an important role in the foundation of a cost-efficient, low carbon dioxide emission economy.
4. Operation: In the whole area of the stadium, no-running-water urinals were placed in the men’s washrooms. Running water was limited with timers at the taps, and the thermostat was set to the minimum for producing hot water. Electronic hand dryers are installed in all the washrooms for minimalizing paper waste. With a system filtering chlorine dioxide, clean water is provided in the whole stadium. With a system optimizing electricity consumption, the use of energy has been decreased by an average of 20% in the stadium. Sports lamps and fluorescent lamps were fitted with LED bulbs and unnecessary electricity use was also minimalized with the help of motion sensors [87]. They use biodegradable plastic bags in their shops. The heating is switched off in any currently empty or unused rooms in the building.
5. Office work: The comprehensive selective waste collection was introduced in 2007. The environmental awareness of the staff is being raised with the help of information leaflets and lectures, and the internal information system and posters also provide information in this field. Employees are encouraged to use minimal electricity (e.g., turn off the monitors, turn off the lights) and use public transport. Moreover, showers are available at the offices, making it possible for the staff to arrive at the workplace running, walking, or by bike. In work areas, the staff can also add their comments, experiences, and suggestions about what provisions could be initiated to make the operation of the club greener. Old printers, printer cartridges, computers, and monitors are recycled at all times. In order to avoid using unnecessary paper and plastic cups, employees use their own cups. Envelopes and copy paper are made of recycled material. Timers assure PC-s, lighting, and screens are turned off for the night and the weekend [87].
6. Transport: Following the proposal of the Premier League, Arsenal began to promote public transport [88], and as a result 70% of supporters do not travel to club events by car. Further developments include more than 7.5 million pounds worth of investment and the expansion of the capacity of underground stops. In addition, they can improve bus, bicycle, and walking facilities by providing over 50 million pounds for TFL (Transport for London) [88]. With the support of Arsenal, further developments are realized, such as road surface repair, modernization of lighting, and obstacle clearing [87].
7. Plant based, low-carbon food: As an SRA approved restaurant, a substantial vegan menu is now available including Dirty Beets burger, Three bean Mexican wrap, Sticky Korean glazed vegetable, and Veggie Chili Cheese Fries [86].

The further plans of the club can be summarized as follows [87]:

- By taking advantage of the popularity of the club, conveying messages to fans that encourage them to adopt an environmentally friendly attitude.
- Finding ways to recycle the mowed lawn cuttings.
- Cooperation with the M&E company in favor of a further decrease in the energy consumption and CO_2_ emissions of the Stadium and other Arsenal premises.
- Increasing use of renewable energy resources (photovoltaic systems, wind energy).

#### 4.3.2. Real Madrid C.F.

Presenting Real Madrid C.F.’s (RM) socially and environmentally related CSR activities, we examined the Corporate Social Responsibility and Sustainability Report of the club (2017–2018) and some articles and case studies about the subject. We found detailed information about their socially related CSR activities, which we are introducing, based on six areas.

1. Social sports schools around the world: The club underlines that their principle is education in values through sport: This is the main means of social action. Through football and basketball as social sports, they promote healthy lifestyles and sport as an outstanding way for children to spend their free time and to work together with families to give them all-round training for over 6000 children between the ages of 5 and 17, in a total of 65 social sports schools. Different programs are available through the schools; for example, (1) the club took part in several inclusive days: in the International Disability Day, the World Autism Day; (2) throughout the season, around 40,000 children and young people participated over 300 international clinical lectures in 43 countries on all five continents. Moreover, 177 of these clinics were held at Real Madrid City, where more than 5000 players from over 35 countries were present [158]. (3) In our country (Hungary) over 70 students have been participating in the “Real after school” sports program. The participation and involvement of students goes far beyond the school activities, as they also enter various social tournaments [181].
2. Social-sports projects with other groups at risk of exclusion: The social-sports schools program is targeted at children (mixed teams of boys and girls) between the ages of 5 and 18 in Europe who are in challenging socio economical situations, which are severe enough to badly affect their education because of poverty and the lack of learning opportunities, or who face different risks or behavioral problems. The RMF (Real Madrid Foundation) works in more than 100 countries. Here, the focus is on European social-sports schools in five different countries: Romania, Italy, the United Kingdom, Portugal, and 14 social-sports schools in shelter homes in Madrid (Spain) [182]. Including the Homeless People group among those being privileged to be involved in the RMF’s activities has been one of the cornerstones in the past few years. Three training groups were established in cooperation with the Spanish Red Cross, Madrid City Council, and the P. Garralda Foundation. This new venture helps more than 50 adults to recover their mental health. Last year, 22 football and 21 basketball events were held involving 2500 prison inmates to orient them to re-education and reintegrate them into society. The program pays special attention to groups in Hospitals, Prisons, Shelters, and Detention Facilities, conveying and reaffirming values for the participants to carry throughout their lives [183].
3. The RM Foundation—an international organization: The international programs of the Foundation in cooperation with some of the key NGOs supporting children have carried on their operations of consolidation, accomplishing altogether over 300,000 h of sports and related activities in almost 2000 working groups, involving 800 teacher-trainers in 300 projects in 76 countries in the 2016/17 season. Over 39,000 children and young people learn about sporting values through these schools and also receive medical care, food, schooling, and more than 200,000 h of social assistance every year [158].
4. Communication, events, and institutional activities: The Real Madrid Foundation has served society through different subject activities in the past few years. As it was celebrating its 20th anniversary, the whole season was full of special events. For example, as the usual Christmas campaign finished, they started the New Year with the second Popular Game of the Foundation, in which 6000 runners participated. Interesting cultural and sports programs made the whole year colorful and eventful.
5. Appearances of players: The current and former players, the ambassadors of the club, appear in several events. Their aim is to support, enhance, and set an example to the young generations. For example, the international school students who visit Madrid have the chance to shake hands with the first team players in a short meeting, which could represent a life-time experience for some and could motivate them to keep up their hard work. Players visit young people with serious illnesses on a weekly basis. Ambassadors for Real Madrid (e.g., Roberto Carlos, Julio Baptista, Julio César, Álvaro Arbeola) contribute to different charity events with their presence.
6. RM Graduate School European University activities: The school has institutions in 12 countries: Spain, Costa Rica, Ecuador, Mexico, Chile, Colombia, Brazil, Peru, Germany, Portugal, the United Kingdom, and Australia. They specialize in the areas of health, sport, management, and communications and offer 15 master’s degrees [184]. The success of the CSR activities of the club can be seen from the fact that the RMF has become involved in academic and international fields in the industry, such as the World Football Summit and Football is More; they have also taken part in conferences concerning team sports and autism at the European University and at the United Soccer Convention and the 4th FIFA Congress on social action. The Madrid Regional Government awarded the club the 2017 Childhood Recognition award; they also won the Montevideo Cervantes School award, the Nine Values Cup, the Olavidia Heart prize, and the YPO Latam Best Business Foundation award.

Reviewing socially related CSR activities, it is clear that they are launching international projects in more and more fields and with the help of their ambassadors—ideal for young people—their goal is to reach the widest possible sections of society through their educational, sports, and charitable programs. They repeatedly emphasize the role of the RMF in these areas, which shows the strengthening of CSR at the club, as they already deal with their CSR activities in the form of a separate organization.

In the following, we will examine how Real Madrid is incorporating environmentally friendly activity in their movement. The first season in which RM had an Environment Division for the whole season was in 2018/2019. The club has been emphasizing a variety of actions to decrease the impacts of its activities on natural resources:

1. Land protection: RM announced on their official pages (2018) that over 200 trees have been planted and chemical products eliminated from the maintenance of the garden areas, and there has also been a reasonable reduction in the use of fungicides on the natural grass football fields. In the 2018/2019 season, ultraviolet lights were used for all the natural grass pitches as a means to prevent plant diseases and avoid having to use chemical substances. At the same time, a biological control program is being run both on the sports pitches and the ornamental gardens with use of their own machine to produce compost tea [159].
2. Waste management: Since 2007, RM has been cooperating with Ecoembes Spain, S.A., to operate selective collection and recovery of light containers and cardboard at the Santiago Bernabéu Stadium and at Real Madrid City, which meant a system was set up for the collection, transport, and subsequent treatment of waste. As a result of this agreement, in the 2018/2019 season as much as 522,520 kg of waste was collected from their facilities, from the Santiago Bernabéu Stadium site, “La Esquina del Bernabéu” shopping center, and from Real Madrid City. This consists of 87% recyclable lightweight containers, paper, and cardboard, and only 13% unsuitable waste. In the 2018/2019 season, 303 tons of light containers and 156 tons of paper and cardboard were recovered through this process. Waste recycling brings the opportunity to make significant cost reductions in scarce raw materials. With these 459 tons of recycled materials made up of containers and cardboard generated in the Real Madrid facilities, it has been possible to make many different products commonly used in society. With recycling, they have been able to significantly reduce energy and water consumption, and greenhouse gas emissions into the atmosphere. This successful approach to lightweight container waste has made it possible to save: (1) 35 tons of CO_2_ emissions, equal to the emissions produced by 103 vehicles. (2) 332,000 KWh of electrical energy consumption. (3) 7,853,779 L of water, equal to the daily consumption of 55,308 people. The new Adidas strip for 2018/2019 for the Real Madrid team was made of plastic recovered from the oceans [156].
3. Office work: Their daily production of printed materials was reduced and replaced with digital publications (from 6179 per month to 2350). The new office building has a certified energy consumption of 241 KWh/m^2^ per year and gas emissions of just 41 Kg CO_2_/m^2^ per year, with 100% being generated by renewable energy resources. During the 2018/2019 season [159], an intelligent call management system was introduced in the lifts in the corporate office building. It manages the allocation according to the traffic at any time, so not all the cabins are running simultaneously, which manages waiting times and streamlines the up/down flows, and the stops at the floors, consequently leading to energy savings.
4. Energy: In 2016, RM ran an Energy Audit of all its buildings, all of which proved to be clean. Here, we mention only some of the measures they have implemented: LED technology in emergency lighting for stands and buildings; motion sensors in public toilets in the stadium and RM City; installation of chargers for electric vehicles at the new corporate office building; frequency inverters in both pumping equipment and in air-conditioning fans. RM are planning to modernize the Santiago Bernabeu by 2022 at a cost of €525 million, which is already approved [157]. A guarantee certificate gives them a green light as regards the sourcing of electrical power supplies. One hundred per cent of the electricity required for their buildings was previously generated by renewable energy sources (mainly solar and wind energy) [158]. In a RM report (2019), it was stated that improvements in the remote management and monitoring of equipment are implemented to minimize their hours of operation, especially regarding the programmed time settings of air conditioning equipment on the basis of the actual levels of occupation of the area. Twenty-two new, electrical cleaning machines have been bought, which can eliminate the storage and use of fossil fuels and almost completely eliminate noise pollution during their use. The use of electrical vehicles is promoted with the installation of six recharging points in the new building car parks.
5. Water management: The annual water consumption of their football fields and the ornamental garden areas of Real Madrid City comes entirely from the recycled water network of the Madrid City Council [158]. In addition, irrigation water is being treated with a magnetic system to cut down on water consumption while producing the same result in terms of irrigation, and all the gardeners receive training and manual sensors to manage irrigation, as part of an irrigation management and control system targeted at maximizing efficiency in water use [159]. Three other systems make the buildings even more modern: heat recovery systems and CO_2_ level sensors using the ventilation systems only when absolutely necessary, lift installations with frequency variators, and quadruplex calling to save waiting times and generate shorter lift journeys.

All these measures resulted in Real Madrid being awarded the STMA—Sports Turf Management Association environmental certification valid until 2020. To sum up, we can say that RM is clearly making efforts to enforce responsible behavior and environmental protection. They have special consideration for society and are a pioneer in different environmental management tools in the Primera División. There were ample information, reports, and case studies to be found about these activities.

## 5. Discussion

More and more examples can be found of the manifestation of the responsible attitude of the sports sector in the past few years. Certain sports, such as football in Europe or basketball and baseball in North America, lead this field and set an example for the sports society.

When analyzing the green activities of the clubs of the Premier League, we found that in most cases no information on sustainability or the environment was available on the websites.

Some information was published in club news and through third party homepages. At only 4 clubs did we see environmental or sustainability reports on the subject, and one team published their green activities as part of the annual report. At the same time, the fact that it was easy to find information on the subject shows that the clubs are actually committed to environmental protection. We found numerical, detailed information on the actions of the clubs, and in some cases, they even announced further sustainability targets.

In the 2010/2011 season, Jenkins [14] examined the environmental sustainability activities of the Premier League’s clubs. Our findings correspond to those in this study, meaning that football clubs do impact on the environment in a negative way; the clubs’ management do recognize this, and work on reducing impacts in fields such as energy, water, waste, land use, and transport. There is also another side of the picture: Football clubs do have a positive effect on the environment as well: through the activities they offer. Having compared the data, it can be concluded that progress has been made in the past few years, the clubs are more and more concerned with the subject and the number of activities has grown, as well.

In the 2016/17 season, Jager—Fifka [185] analyzed CSR practices in English and German football clubs. Their results were based on 24 expert interviews (13 German, 11 English). When asked about environmental sustainability, most CSR managers (*n* = 8/11) stated that it is not high on the agenda. In contrast, according to the documents analyzed, we can see that the importance of this area has increased, as is described by the clubs in the form of a number of specific measures. There is also likely to be a kind of mindset change in clubs, since it is not just the social aspect of CSR that is considered dominant anymore

BBC Sport has worked with the United Nations-backed Sport Positive Summit [186]. In 2019, for the first time, Sport Positive systematically harmonized key environmental sustainability data for each Premier League football club. The categories reflect the environmental impacts a football match causes, and the initiatives that are being run at clubs [86].

All in all, the results indicate that clubs have growing ambitions and active initiatives related to different aspects of environmental sustainability.

Investigating the environmental protection operations of the Primera División’s clubs, it was found that the only team with environmental reports is Real Madrid CF (Corporate Social Responsibility and Sustainability Report), publishing a document which closely reviews the actions taken to benefit society and the environment. As far as the other clubs are concerned, with most of them no sustainability or environment page was available on the websites. Some information was communicated in the club news and through third party websites; however, we could not find any information for most clubs, either in English or Spanish. This does not mean that these clubs have no environmental initiatives, but that they pay less attention to publishing them. For most clubs, the available information is rather superficial, only mentioning operations without any detailed and exact descriptions. Some clubs have distant targets in the field of environmental protection, which were published two or three years ago; at the same time, there is no information on their actual realization.

On 25 September 2015, world leaders agreed on global aims as part of the sustainable development agenda in order to eliminate poverty, protect the planet, and provide welfare for everyone. Seventeen profound aims were articulated [187], with further subfields, which are to be fulfilled in the next 15 years. Some clubs present their responsible attitude adjusted to these, but no exact description or statistical targets are introduced; therefore, it is rather hard to obtain information on the extent of the clubs’ actual commitment to them and their realization.

The promotion of the green activities of the La Liga is corroborated by the fact that the COP25 Climate Change Conference (United Nations Climate Change Conference) was held in Madrid in 2019 and was supported by La Liga as part of the league’s goals to build sustainability. La Liga enhanced the conference by implementing a number of actions during matchdays [143].

In the last two years, La Liga and its Foundation have been involved in the Fair Play Social project, which aims to apply social responsibility practices throughout the current business management model of the institution. A total of 22 clubs joined the program with a common purpose of harmonizing their organizational, environmental, and social activities with sustainability programs. With the analytic methods employed, we obtained a significantly deeper understanding of a holistic view of sustainability aspirations. Five key areas were determined, which serve as a basis for modelling club activities in terms of sustainability (awareness campaigns, waste management systems, reduction of carbon footprint and offsetting, partnerships with environmentally committed companies, and responsible energy use) [144].

As a consequence of the abovementioned cooperation, it is highly likely we will see some progress in the field of realization and publication of the green endeavors of the clubs in the coming years.

Comparing the environmental protection measures of the two Leagues, it turns out that currently the clubs of the Premier League are further ahead in green activities and these are also widely published. However, it can also be concluded that La Liga and its clubs do make efforts to implement environmentally friendly operations to make sure football becomes an ever more sustainable industry.

Investigating the socially related CSR activities of the Premier League, we found that clubs operate such measures through foundations. We learnt about the foundations, their goals and targets, their major guidelines and several related activities. These operations are presented in detail, and there were no difficulties in finding information connected to our research field. The practical application of these efforts has become well established in the past few years, and they can address an ever-wider section of society through their various programs. Understanding how privileged their situation is, clubs are grateful to their supporters and invest in CSR to give some of the support back in return. Educational and sports programs appear to be the most popular. The quality of life is the third field related to CSR activities which they concentrate on (social inclusion, cultural integration, health programs, disability sports). Over 100 socially related CSR activities are carried out at the clubs annually. Our findings are similar to Jager—Fifka [185]. In 2016 they noted that all English interviewees (n = 11/11) mentioned physical and mental health and educational programs, which were in the public eye at that time. A majority also mentioned issues such as social inclusion, participation, employability, or sports development. Rosca [15] analyzed what kind of CSR activities English football clubs are implementing. He determined 7 fields in order to classify CSR activities (charity, health, family, cultural, social, sport, and educational programs). He claims that the quantity and the quality of the CSR activities may depend on the wealth of clubs or on the role the club plays in its community. In addition to this, the size of the city and its neighborhoods can be the reason why its football clubs are so involved in the community. According to Breitbarth and Harris [188], the integration of CSR into football creates additional value for stakeholders, while their close relations with the local community require them to be active members of society.

In La Liga, the great majority of the clubs operate their social activities through foundations, and many of the clubs present information on their websites. At one third of these clubs, the information can be accessed only in Spanish, limiting the circle of those who can access this information. The amount and detail of the information is, however, rather varied. In some cases, we can only find guidelines about what the clubs prioritize, but we do not find much or any detailed examples of them. In some cases, we can find out about activities carried out over many years. We can conclude likewise that educational programs are the most emphasized, followed by social and health initiatives. Ruiz-Mora and Guerrero-Navaro [189] analyzed what kind of CSR activities Spanish top clubs are most involved in. They highlight education and personal development, then social assistance, families and cooperation, then research, technology and culture, and finally environment and employees. These results also show that environmental protection receives much less attention than the social aspects of CSR. According to PWC [190], during the 2016–2017 football season, LaLiga contributed 62.8 million euros to CSR projects, almost 4 times more than what the Bundesliga provides and more than 1.5 times what the Premier League donates. For every 100 euros donated by LaLiga, 1.72 is invested in CSR projects. That is, more than 2 times the average investment ratio of the main European football leagues and more than 15 times the average contribution of the business world.

It is not usual to find material on environmentally and socially related CSR activities in the football clubs we researched. This could be partly due to a lack of activities and resources, both human and financial, and to a lack of CSR reporting practice. Nevertheless, we have experienced an increasing tendency within the corporate sector to publish on social/environmental activities, and it is probable that the sports industry will see similar expectations to do so in the future. Even though environmental issues are a more and more fundamental aspect of CSR, it seems that currently not all of the football clubs we analyzed are involved in environmental action. Football clubs are much more preoccupied with community and employee stakeholder groups. There are perhaps historical reasons for this. Football clubs have long been seen as part of the community, and policy makers are finding that football clubs can be a means of delivering broader policy objectives around health, education, social cohesion, and charitable donations.

By introducing case studies, we aimed to present detailed examples to follow of various CSR activities for sports society. This is significant, because if we overview the environmental efforts applied by the sports sector, it can be seen that today only isolated solutions are present, the effects of which are insignificant compared to the extent of the global environmental burden. On the other hand, by promoting exemplary good-practices, considering their environmental-economic benefits, and thanks to the effects of the synergy and taking advantage of positive external influences, within a few years the impact of these activities will be tangible.

The presence of socially related CSR activities is more widespread in practice and is more common among the activities of the clubs. It can be seen in relation to the two case studies that the clubs adopt several practices, addressing wide social groups, involving players and other professionals of the club. These practices can serve as a guideline for clubs where CSR activities are not yet emphasized and can certainly be adapted in other sports as well. In the interests of realizing the abovementioned goals, a broad collaboration of sports society is necessary, and thus, the integration of CSR solutions into sports can be effective and successful.

To answer our research questions, it can be said that clubs’ CSR related issues can clearly be divided into 2 categories: socially and environmentally related CSR activities. The predominance of measures taken to serve society can be observed; currently, more emphasis is placed on the introduction of these measures by clubs, and the practice of this is better developed.

However, we can also see from the documents examined that clubs are integrating more and more environmental aspects into the greening of their operations. For the clubs of the two leagues, waste management and water/energy efficiency are the areas where more activity can be observed, but we can also observe green efforts in the areas of clean energy, land use and landscape, and the environmental impact of sports-related activities. We found less or no information in these latter areas, but that does not mean these areas do not belong closely to the greening of a club’s operations. Perhaps the range of good practices that can be applied in these areas is even less developed, although they will gradually incorporate new environmental aspects into their operation, but it is expected that progress will be observed in these areas as well.

The socially related CSR activities are diverse, but basically fall into 4 categories: sport and health/educational/social and cultural/charity programs. According to the documents analyzed, it was clear that in addition to serving the local community, they are also involved in more and more international programs, both in the field of sports and in education. In addition to football, they also offer opportunities to practice many other sports for different ages. They also provide programs for vulnerable groups in society, with a strong emphasis on integration. Their charity programs also cover the fields of sports, healthcare, and food.

## 6. Conclusions

The sports world is also going through a period of transformation towards genuinely sustainable progress. Football is a brilliant form of communication in European society and in the wider world. Football has the potential to be the perfect platform to raise inclusion in sustainability programs. The concept of “paying society back for what it has given” is key in the world of football and must be present in all the areas in which the industry has a footprint around the planet, including the environment.

To sum up, it is necessary to examine CSR activities in sport, which is becoming an increasingly popular research area. Sport as a global industry has to undertake its share of the responsibility to create a wide social unity, with sustainable development pinned on its flag. In activities to promote sustainability, all players in the sports sector—athletes, associations, the organizers of sport events, and the representatives of economic and social organizations related to sport—need to participate.

## Figures and Tables

**Table 1:** Clubs in the Premier League and Primera División in the 2018/2019 season.

Primera Division	Premier League
Alavés	AFC Bournemouth
Atlético de Madrid	Arsenal
Athletic Bilbao	Aston Villa
Barcelona	Brighton and Hove Albion
Celta Vigo	Burnley
Eibar	Chelsea
Espanol Barcelona	Crystal Palace
Getafe	Everton
Granada	Leicester City
Leganés	Liverpool
Levante	Manchester City
Mallorca	Manchester United
Osasuna	Newcastle United
Real Betis	Norwich City
Real Madrid	Sheffield United
Real Sociedad	Southampton
Real Valladolid	Tottenham Hotspur
Sevilla	Watford
Valencia	West Ham United
Villarreal	Wolverhampton Wanderers

Source: [66,67].

**Table 2:** Environmental protection activities of the teams in the Premier League.

	Land Use and Land Scape	Clean Energy	Energy Efficiency	Water Efficiency	Waste Management	Certification	Environmental Impact of Activities Deriving from Sport		
							Transport	Food	Office Work
Arsenal	X	X	X	X	X		X	X	X
Aston Villa		X	X	X	X		X	X	
AFC Bournemouth			X	-	X		X	X	
Brighton and Hove Albion		X	X	X	X	BREEAM-very good	X	X	
Burnley		X	X	X	X		X	-	
Chelsea		-	X	X	X	BREEAM-excellent	X	X	X
Crystal Palace			X		X		X	X	
Everton		X	X	X	X		X		
Leicester City			X	X	X	developing plan: ISO14001	X	X	
Liverpool			X	X	X	ISO500001	X	X	
Manchester City		X	X	X	X	LEED-gold	X	X	
Manchester United	X	X	X	X	X	ISO 14001	X	X	X
Newcastle United		X	X	X	X		X	X	
Norwich City		X	X	X	X		X	-	
Sheffield United		X	X	X	X		X	X	
Southampton			X	X	X		X	X	
Tottenham Hotspur		X	X	X	X		X	X	
Watford		X	X	X	X		X	X	
West Ham United		X	X	X	X		X	X	
Wolverhampton Wanderers			X	X	X		X	X	

Source: [86,87,88,89,90,91,92,93,94,95,96,97,98,99,100,101,102,103,104,105,106,107,108,109,110,111,112,113,114,115].

**Table 3:** Socially related CSR activities of the English teams.

	Educational Programs	Sport/Health Programs	Social/Cultural Programs	Charity Programs
Arsenal	X	X	X	X
Aston Villa	X	X	X	X
AFC Bournemouth	X	X	X	X
Brighton and Hove Albion	X	X	X	X
Burnley	X	X	X	X
Chelsea	X	X	X	X
Crystal Palace	X	X	X	X
Everton	X	X	X	X
Leicester City	X	X	X	X
Liverpool	X	X	X	X
Manchester City	X	X	X	X
Manchester United	X	X	X	X
Newcastle United	X	X	X	X
Norwich City	X	X	X	X
Sheffield United	X	X	X	X
Southampton	X	X	X	X
Tottenham Hotspur	X	X	X	X
Watford	X	X	X	
West Ham United	X	X	X	X
Wolverhampton Wanderers	X	X	X	

Source: [95,104,111,116,117,118,119,120,121,122,123,124,125,126,127,128,129,130,131,132,133,134,135,136,137,138,139,140,141,142].

**Table 4:** Environmental activities by the clubs of the La Liga.

	Land Use and Land Scape	Clean Energy	Energy Efficiency	Water Efficiency	Waste Management	Certification	Environmental Impact of Activities Deriving from Sport		
							Transport	Food	Office Work
Alavés									
Athletic Bilbao		X	X	X	X	LEED	X		
Atletico de Madrid		X	X	X	X				
Barcelona	X	X	X	X	X	DGNB pre-certification	X		
Celta Vigo	X		X	X	X	ISO 14001			
Espanol Barcelona									
Getafe			X	X	X				
Granada									
Leganés					X				
Levante									
Mallorca					X				
Osasuna									
Real Betis	X	X	X	X	X	signed up to the United Nations Climate Neutral Now			
Real Madrid	X	X	X	X	X	UNE EN ISO 14064-3:2012; UNE 171330-2014			X
Real Sociedad					X				
Real Valladolid									
Sevilla		X	X						
Valencia					X				
Villareal	X		X						

Source: [143,144,145,146,147,148,149,150,151,152,153,154,155,156,157,158,159,160].

**Table 5:** Socially related CSR activities of Spanish clubs.

	Educational Programs	Sport/Health Programs	Social/Cultural Programs	Charity Programs
Alavés		X	X	X
Athletic Bilbao	X	X	X	
Atletico de Madrid	X	X	X	X
Barcelona	X	X	X	X
Celta Vigo	X	X	X	X
Eibar	X	X	X	X
Espanol Barcelona	X	X		X
Getafe	X	X	X	X
Granada	X	X	X	X
Leganés	X	X	X	X
Levante	X	X	X	X
Mallorca	X	X	X	X
Osasuna	X	X	X	X
Real Betis	X	X	X	X
Real Madrid	X	X	X	X
Real Sociedad	X	X	X	X
Real Valladolid	X	X	X	X
Sevilla	X	X	X	X
Valencia	X	X	X	X
Villareal	X	X	X	X

Source: [158,159,161,162,163,164,165,166,167,168,169,170,171,172,173,174,175,176,177,178,179].
